# The Therapeutic Potential of Two Egyptian Plant Extracts for Mitigating Dexamethasone-Induced Osteoporosis in Rats: Nrf2/HO-1 and RANK/RANKL/OPG Signals

**DOI:** 10.3390/antiox13010066

**Published:** 2024-01-01

**Authors:** Samar R. Saleh, Omnia M. Saleh, Ashraf A. El-Bessoumy, Eman Sheta, Doaa A. Ghareeb, Saber M. Eweda

**Affiliations:** 1 Biochemistry Department, Faculty of Science, Alexandria University, Alexandria 21515, Egypt; omnia.mohamed_pg@alexu.edu.eg (O.M.S.); ashraf.elbasomy@alexu.edu.eg (A.A.E.-B.); d.ghareeb@alexu.edu.eg (D.A.G.); seweda@taibahu.edu.sa (S.M.E.); 2 Bio-Screening and Preclinical Trial Lab, Biochemistry Department, Faculty of Science, Alexandria University, Alexandria 21515, Egypt; 3 Pathology Department, Faculty of Medicine, Alexandria University, Alexandria 21515, Egypt; iman.sheta@alexmed.edu.eg; 4 Department of Clinical Laboratory Sciences, College of Applied Medical Sciences, Taibah University, Madinah 42353, Saudi Arabia

**Keywords:** purslane (*Portulaca oleracea* L.), chicory (*Cichorium intybus* L.), natural medicine, bone resorption, alendronate, NFATc1, glucocorticoids, antioxidant, inflammation

## Abstract

The prolonged use of exogenous glucocorticoids, such as dexamethasone (Dex), is the most prevalent secondary cause of osteoporosis, known as glucocorticoid-induced osteoporosis (GIO). The current study examined the preventative and synergistic effect of aqueous chicory extract (ACE) and ethanolic purslane extract (EPE) on GIO compared with Alendronate (ALN). The phytochemical contents, elemental analysis, antioxidant scavenging activity, and ACE and EPE combination index were evaluated. Rats were randomly divided into control, ACE, EPE, and ACE/EPE MIX groups (100 mg/kg orally), Dex group (received 1.5 mg Dex/kg, Sc), and four treated groups received ACE, EPE, ACE/EPE MIX, and ALN with Dex. The bone mineral density and content, bone index, growth, turnover, and oxidative stress were measured. The molecular analysis of RANK/RANKL/OPG and Nrf2/HO-1 pathways were also evaluated. Dex causes osteoporosis by increasing oxidative stress, decreasing antioxidant markers, reducing bone growth markers (OPG and OCN), and increasing bone turnover and resorption markers (NFATc1, RANKL, ACP, ALP, IL-6, and TNF-α). In contrast, ACE, EPE, and ACE/EPE MIX showed a prophylactic effect against Dex-induced osteoporosis by modulating the measured parameters and the histopathological architecture. In conclusion, ACE/EPE MIX exerts a powerful synergistic effect against GIO by a mode of action different from ALN.

## 1. Introduction

Osteoporosis is a prevalent metabolic bone disease characterized by decreased bone mass, altered bone architecture, increased bone fragility, and increased fracture risk [1,2]. Osteoporosis affects one out of eight men and one-third of women over 50 years [3]. Because key warning signals, including spinal fractures, deformations, height loss, and disabilities, do not manifest before fractures occur, osteoporosis is a silent illness [4]. Osteoporosis is classified as primary osteoporosis, which includes postmenopausal osteoporosis, and secondary osteoporosis, which includes a well-known medicine called glucocorticoids (GCs) [1]. 

Glucocorticoids are considered anti-inflammatory and immunosuppressive and are used to treat inflammatory conditions such as rheumatoid arthritis and systemic lupus erythematosus [5]. Although normal concentrations of GCs encourage osteoblast development, pharmaceutical dosages hinder osteoblastogenesis. Prolonged usage of GCs is linked to side effects, such as glucocorticoid-induced osteoporosis (GIO), which is the most prevalent type of secondary osteoporosis. GIO is brought on by consuming synthetic GCs in large doses over an extended period [6,7]. GCs impede the creation of new bone and encourage bone resorption, directly and indirectly, repressing bone growth [7] indirectly by decreasing calcium (Ca) absorption from the intestine and increasing parathyroid hormone (PTH), and directly by inhibiting pro-osteoblastic genes like the important pro-osteogenic transcription factor osteocalcin (OCN), increasing osteoblast apoptosis and boosting osteoclast proliferation [6].

Additionally, GCs encourage bone resorption through the production of receptor activators of nuclear factor kappa-B ligand (RANKL) and cytokines, including tumor necrosis factor-alpha (TNF-α) and interleukin 6 (IL-6) [8]. RANKL stimulates osteoclast formation through NF-κB activation via IκB kinase (IKK) phosphorylation and activation [9]. A triad composed of receptor activators of nuclear factor kappa-B (RANK), its ligand (RANKL), and osteoprotegerin (OPG) is essential for bone remodeling and homeostasis [10,11]. RANKL/RANK binding activates the transcriptional nuclear factor of activated T cells of type 1 (NFATc1), which boosts the expression level of osteoclastogenic factors, such as acid phosphatase (ACP), which promotes osteoclastogenesis and bone resorption [12,13]. On the other hand, OPG is secreted primarily by osteoblasts and acts as a soluble decoy receptor of RANKL. The OPG/RANKL interaction prevents RANKL/RANK binding and inhibits osteoclast formation and bone resorption. The RANKL/OPG ratio indicates bone health and reflects the balance between bone formation and resorption [11]. GCs directly stimulate osteoclast differentiation by increasing RANKL/RANK signaling and OPG inhibition [14,15]. 

Moreover, synthetic GCs like dexamethasone (Dex) might worsen oxidative stress, which is a major cause of osteoporosis, by boosting the generation of free radicals or suppressing the activity of antioxidants [16]. Reactive oxygen species (ROS) are crucial in promoting osteoclast formation, while the osteoblasts’ antioxidant molecules hinder ROS’s effects. The increased ROS formation disrupts the balance between osteoclasts and osteoblasts, leading to increased bone resorption, bone formation inhibition, and bone mass loss [17]. The nuclear factor erythroid 2-related factor 2 (Nrf2), widely expressed in osteoblasts, osteoclasts, and other bone cells, is a vital transcription factor for endogenous antioxidant enzymes [18]. Nrf2 plays an essential and controversial role in the defense against oxidative stress and regulation of bone homeostasis. Nrf2 inactive form is constitutively expressed in the cytoplasm, and its accumulation and activation in the nucleus are favored in oxidative injury [16,19]. Under oxidative stress, Nrf2 liberated from its inhibitory form and subsequently translocated to the nucleus and attached to the antioxidant-responsive element (ARE) regions in the promoter of different antioxidant genes, leading to increased expression of diverse detoxification and antioxidant enzymes as heme oxygenase-1 (HO-1) [20]. It has been shown that Nrf2/HO-1 deficiency decreased bone density during bone remodeling, promoted RANK ligand expression, and induced osteoclastogenesis and bone resorption [21,22]. Consequently, the Nrf2/HO-1 axis has a potential role in promoting osteoblast differentiation and inhibiting osteoclastogenesis. However, the role of Nrf2 is controversial, where activation of Nrf2 directly inhibits osteoclast differentiation by fighting oxidative stress. Other studies explained that Nrf2 is indispensable for acquiring and maintaining bone mass even under various stress conditions. More studies show that Nrf2 overactivation inhibits bone formation, while its moderate activation promotes increased bone mass [18,19,23]. Earlier research reported that GCs induced osteoporosis through Nrf2/HO-1 signaling disruption [16,24]. Overall, many patients with anticipated chronic GC exposures should start anti-osteoporosis pharmacotherapy.

Since ancient times, biologically active compounds from natural resources have been of great interest for their application in many non-infectious diseases such as osteoporosis [25]. Secondary metabolites are the most prominent bioactive components found in such plants and are primarily responsible for these plants’ biological activity [26]. Chicory (*Cichorium intybus* L.) and purslane (*Portulaca oleracea* L.) are edible Egyptian plants considered natural exogenous sources of biologically active compounds such as antioxidants [25]. Chicory is a perennial herb that belongs to the *Asteraceae* family’s Cichorium genus. [26]. Chicory roots contain a variety of bioactive compounds, including alkaloids, flavonoids, phenolics, inulin, tannins, saponins, sesquiterpene lactones, chlorophyll pigments, unsaturated sterols, and coumarins, that are beneficial to humans [27,28]. Chicory root water extract was reported to contain large amounts of polyphenols, resveratrol, and naringin. Chicory exhibits a wide range of biological effects, including anti-inflammatory, antioxidant, hepatoprotective, immune, cardiovascular, hypolipidemic, antidiabetic, anticancer, gastro-protective, antibacterial, and many others [26,28]. Due to its high inulin concentration, chicory promotes calcium absorption, increases bone mineral content (BMC), and slows the loss of bone mineral density (BMD) [29]. 

Purslane is a medicinally important plant considered a perennial herb belonging to the *Portulaca* genus (40–100 species), *Portulacaceae* family [30]. Purslane leaves are a potential source of a variety of bioactive compounds for humans, including phenolics, alkaloids, coumarins, glycoside, high levels of omega-3 fatty acids, and flavonoids such as apigenin, kaempferol, quercetin, luteolin, and myricetin. [28,31]. The biological properties of purslane include anti-inflammatory, antioxidant, antiseptic, antispasmodic, diuretic, vermifuge, anti-scorbutic, antibacterial, hepatoprotective, immunological, hypolipidemic, antidiabetic, gastro-protective, and others [28]. 

A pharmacological study on the activity of purslane and chicory extracts and their possible mechanism in preventing pathological bone destruction has not been well defined yet. This study aims to investigate the management of GIO by combining chicory root aqueous extract (ACE) and purslane leaf ethanolic extract (EPE), compared to their individual use and bisphosphonate treatment. This will be through studying the RANK/RANKL/OPG, NF-κB /IKK and Nrf2/HO-1 Pathways.

## 2. Materials and Methods

### 2.1. Chemicals and Kits

Dexamethasone (Dex), aluminum chloride, folin–ciocalteau reagent, 2,2-diphenyl-1-picrylhydrazyl (DPPH), sodium nitroprusside, Griess reagent (sulphanilamide and N-(1-Naphthyl) ethylenediamine dihydrochloride), tris-HCL, pyrogallol, thiobarbituric acid, trichloroacetic acid, 5,5-dithio-bis-2-nitrobenzoic acid (DTNB), cumene H_2_O_2_, reduced glutathione (GSH), and phenazine methosulfate fluoride (PMSF) were purchased from Sigma-Aldrich (St. Louis, MO, USA). The total protein assay kit was purchased from BioMED (Cairo, Egypt). Total RNA extraction kit, cDNA synthesis kit, and SYBR green with low ROX were purchased from Enzynomics (Daejeon, Republic of Korea). Primers and antibodies were obtained from Willowfort (Nottingham, UK) and Cell Signaling Technology (Danvers, Massachusetts, USA), respectively. Other chemicals were of analytical grades. 

### 2.2. Plant Sources and Preparation of Different Extracts

Chicory roots and purslane leaves were collected from Egyptian farmers in winter (2019) and identified by botany researchers at Alexandria University’s Faculty of Science. Table 1 illustrates plant material collection and extract preparation.

#### 2.2.1. Preparation of Aqueous Chicory Root Extract (ACE)

Chicory roots were gathered, rinsed with water, and dried at room temperature before being lyophilized at −80 °C and ground to a fine powder. The dried powdered root was soaked in water, decanted, filtered, and reduced to 10% of its original volume. Finally, the resulting powder (yield of 10%) was obtained by lyophilization and stored at −20 °C for further use [25,28].

#### 2.2.2. Preparation of Ethanolic Purslane Leaf Extract (EPE)

Purslane leaves were collected, cleaned using water, dried at room temperature, and ground into powder. Five times as much ethanol (70%) was added to the obtained powder. The mixture was filtered and lyophilized after being reduced with a rotary evaporator. Finally, the powder was obtained, yielding about 16% [28,32]. 

### 2.3. In Vitro Analysis for Characterization of ACE and EPE

#### 2.3.1. Total Phenolic Content

Total phenolic content was measured in ACE and EPE based on the Folin-Ciocalteau colorimetric technique [33]. Gallic acid was employed as the reference for measuring the produced blue color using spectrophotometry at 760 nm. Data are expressed as mg equivalent of gallic acid/g extract.

#### 2.3.2. Total Flavonoid Content

Total flavonoid content was measured at 510 nm against a blank reagent using the colorimetric aluminum chloride technique, and quercetin was employed as the standard [34]. Data are expressed as mg equivalent of quercetin/g extract.

#### 2.3.3. Total Alkaloid Content

Total alkaloid content was measured by adding 200 mL of 10% acetic acid in ethanol to 5 g of the extract. The mixture was then covered with aluminum foil and left for two days. The mixture was filtered and reduced to a quarter of its original volume by being heated in a water bath. After that, the solution was precipitated by gradually adding concentrated ammonium hydroxide and then filtered again. The final weight of the residue was determined, and the total alkaloids percentage (%) was calculated [35] as follows:

$$\%\;\mathrm{Alkaloids}\;=\frac{\mathrm{Weight}\;\mathrm{of}\;\mathrm{precipitate}\;\left(g\right)}{\mathrm{Initial}\;\mathrm{weight}\;\mathrm{of}\;\mathrm{sample}\;\left(g\right)}\times 100$$

#### 2.3.4. Inulin Content

Inulin content was determined colorimetrically by resorcinol reaction, which depends upon the hydrolysis of inulin to fructose, giving a condensed colored compound whose absorption is measured at 490 nm. Inulin was used as a standard [36]. Inulin content was expressed as g/100 g extract (g%) using the following equation:

$$\mathrm{Inulin}\;\mathrm{content}\;\left(g\%\right)=\frac{\mathrm{Sample}\;\mathrm{Absorbance}}{\mathrm{Standard}\;\mathrm{Absorbance}}\times \;\mathrm{Standard}\;\mathrm{concentration}\;\left(g\%\right)$$

#### 2.3.5. HPLC Analysis of Phenolic and Flavonoid Compounds

Analysis of phenolic and flavonoid compounds was applied using HPLC-Agilent Series 1100 (Agilent, Santa Clara, CA, USA) as described by Lin, Juan [37] and Kuntić, Pejić [38]. C18 column (125 mm× 4.60 mm, 5 µm particle size) and UV/Vis detector set at 250 nm for phenolic acids and 360 nm for flavonoids were used. Phenolic acids were separated using a gradient mobile phase of methanol and acetic acid in water. Flavonoids were separated by employing a mobile phase of acetonitrile (A) and 0.2% (*v*/*v*) aqueous formic acid (B) with an isocratic elution (70:30) program. The solvent flow rate was 1 mL/min, and the separation was performed at 25 °C. The injection volumes were 25 μL. Phenolic and flavonoid compounds were expressed as (mg/g extract). All chemical standards were HPLC grade (Sigma-Aldrich, St. Louis, MO, USA).

#### 2.3.6. Minerals Analysis in ACE and EPE 

Minerals contents were measured by using Analytik Jena GmbH—contrAA 300—High-Resolution Continuum Source Atomic Absorption Spectrometer, Jena, Germany. US EPA Method 200.7 [39,40] was used. One g of ACE or EPE was digested in a hydrochloric acid/nitric acid mixture (3:1) for one hour at 100 °C. The digested extracts were used for the analysis. Data were expressed as μg/mg extract.

### 2.4. Antioxidant Properties of ACE, EPE, and Their Combination

The extracts of ACE, EPE, and their combination (ACE/EPE MIX) were dissolved in dis.H_2_O (10 mg/mL) for all antioxidant experiments.

#### 2.4.1. 2,2-Diphenyl-1-picrylhydrazyl (DPPH) Scavenging Activity

Following the procedure described by Manzocco, Anese [41], 20 µL of DPPH solution was mixed with 5 µL ACE and EPE to start the reaction. The absorbance was measured at 517 nm after 30 min of dark incubation. The percentage of radicals scavenging was determined.

#### 2.4.2. Ferric Radical Antioxidant Power (FRAP)

The free radical antioxidant power was determined by adding 1ml phosphate buffer (0.2 M) and 1ml potassium ferricyanide (1%) to 1ml extract and incubating at 50 °C for 20 min. Then, 1 mL trichloroacetic acid (10%) was added and centrifugated at 3000 rpm for 10 min. After that, 1 mL of the upper layer was mixed with 1 mL of distilled water and 0.2 mL of ferric chloride [35]. The absorbance was measured at 700 nm. FRAP was expressed as mg/mL.

#### 2.4.3. Nitric Oxide (NO) Scavenging Activity

Nitric oxide scavenging activity was determined by adding 50 µL sodium nitroprusside (10 mM in phosphate buffer, pH 7.4) to 50 µL of ACE and EPE in various concentrations. After 60 min of incubation at room temperature, 100 µL of Griess reagent dissolved in 2.5% phosphoric acid was added. The solution was incubated at room temperature for 30 min. At 540 nm, the absorbance value was determined, and the % of radicals scavenging was calculated [42,43].

The following formula was used to determine the percentage of radical scavenging activities.

$$\%\;\mathrm{inhibition}\;=\frac{A\;\mathrm{control}-A\;\mathrm{sample}}{A\;\mathrm{control}}\times 100$$

where A sample is the extract’s absorbance, and A control is the control’s absorbance.

To calculate the radicals’ 50% inhibition, IC50 values were employed. The values of the mixture (IC50 EPE/IC50 ACE) and the IC50 for each assay of EPE and ACE were calculated. As we previously reported in Saleh, Manaa [28], a variety of ratios were employed to assess the antioxidant activities (1:1, 1:2, 1:4, 3:4). Experimental study approved that A 1:1 (IC50 EPE: IC50 ACE) ratio demonstrated the strongest synergistic impact for the mixture of ACE and EPE extracts. Therefore, this ratio will be used in the in vivo study.

### 2.5. In Vivo Study

#### 2.5.1. Animals

At the animal house of the Institute of Graduate Studies and Research, Alexandria University, Egypt, 64 sexually mature male Sprague Dawley rats were obtained, weighing 155–180 g (from 10 to 14 weeks of age). All rats were kept under a controlled environment in cages (6 rats/cage for control groups and eight rats/cage for induced groups) and nourished with a typical diet and tap water. The Institutional Animal Care and Use Committee (IACUC) at Alexandria University approved all animal studies following the recognized ethical norms for scientific research (AU: 04 21 12 25 1 01, endorsed on 25 December 2021).

#### 2.5.2. Model of GIO-Induced Bone Loss and Treatment

**Figure 1:**
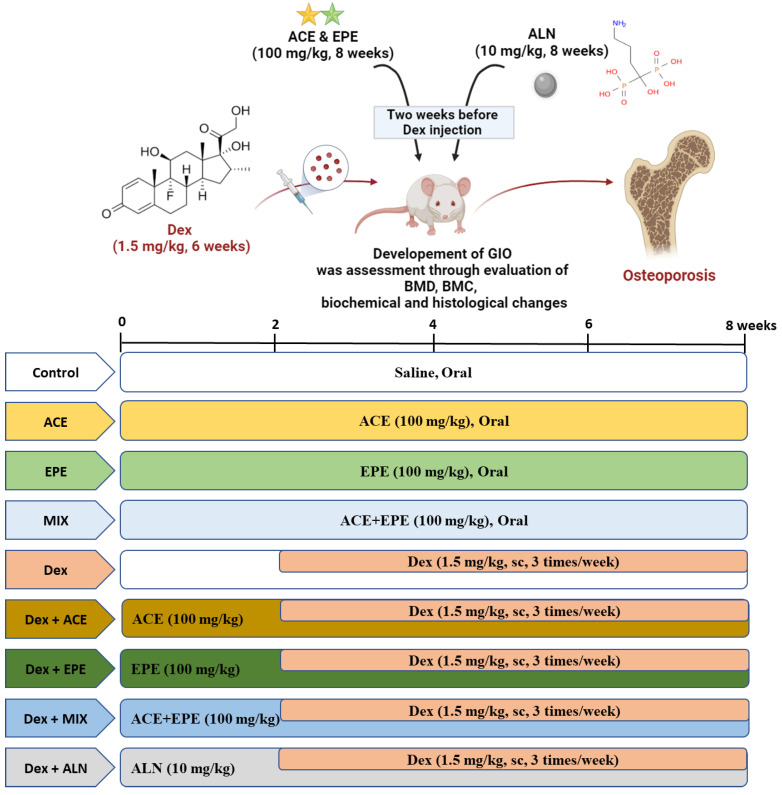
The experimental design and treatments.

After two weeks of acclimatization, rats were randomly allocated into nine groups: 6 rats/group for the four control groups and 8 rats/group for the induced and the four treated groups. Controls were orally administered saline (0.9%), EPE (100 mg/kg) [28], ACE (100 mg/kg) [44], and ACE/EPE MIX based on their IC50 (100 mg/kg, 80/20 mg/kg (ACE/EPE) [28], for 8 weeks. The osteoporosis-induced group received Dex subcutaneously at a dose of 1.5 mg/kg, 3 times/ week, for 6 weeks starting from week 2 of the experimental period [44,45]. Finally, the treated groups received Dex along with EPE (Dex + EPE, 100 mg/kg), ACE (Dex + ACE, 100 mg/kg), ACE/EPE MIX (Dex + MIX, 80/20 mg/kg), and anti-resorptive bisphosphonate, Alendronate (Dex + ALN, 10 mg/kg). These treatments were dissolved in saline solution and administered by oral gavage daily for two weeks before Dex and continued for another six weeks with Dex (Figure 1).

#### 2.5.3. Blood and Tissue Collection and Their Preparation

Rats were starved overnight and sacrificed using the proper anesthesia when this study’s period was terminated. Blood samples were taken through the inferior vena cava. Using centrifugation, serum was isolated (3000 rpm, 15 min, 4 °C) and stored at −20 °C. Muscles and connective tissues were dissected and removed from each animal’s femur. Left femurs were used for bone density measurements and histopathological analysis. The right femurs were washed with normal saline (0.9%) and crushed in liquid nitrogen, and bone marrow was removed and kept at −80 °C until used for molecular and biochemical analyses. 

Phosphate buffer saline (0.1 M, pH 7.4, 1:9 *w*/*v*) was used for bone homogenization. The homogenate was centrifuged, and the supernatant was kept at −80 °C until used to estimate oxidative stress biomarkers.

#### 2.5.4. Bone Mineral Content, Bone Mineral Density, and Bone Index

Dual-energy X-ray absorptiometry (DEXA) was used in the Bone Minerals Density Unit, Medical Service Unit, National Research Center, Dokki, Egypt, to determine the amount of bone mineral content (g) and bone mineral density (g/cm^2^). The bone index was obtained by dividing the femur’s weight by the final body weight [46].

#### 2.5.5. Biochemical analysis

a. Determination of bone formation and bone turnover parameters

Serum levels of ionized calcium (Ca-I) and alkaline phosphatase activity (ALP) were measured using commercial kits obtained from Spectrum, Egypt. Their serum levels were represented as mg/dl and U/L, respectively.

Serum parathyroid hormone, osteocalcin (OCN) levels, and acid phosphatase (ACP) activity were estimated using the kits purchased from COBAS, Roche Diagnostics International Ltd, Germany. Their levels were expressed as pg/mL, ng/mL, and U/L, respectively.

b. Oxidative stress biomarkers

Bone lipid peroxidation:

Malonaldehyde (MDA) is lipid peroxidation’s main secondary byproduct, which was assessed calorimetrically using a Thiobarbituric acid reaction under acidic conditions. At 532 nm, the bone MDA level was obtained as µmol/mg protein [47].

Bone nitric oxide (NO):

Utilizing a quantitative Griess colorimetric test, NO generation was indirectly assessed. This method involves the enzymatic conversion of nitrates (NO^3−^) to nitrites (NO^2−^) using nitrate reductase and NADPH in the presence of oxygen. The determination was assessed calorimetrically by forming a stable azo compound, resulting in an intense purple color, spectrophotometrically measured at 540 nm. The bone NO level was reported as µmol/mg protein [48].

Bone glutathione-s-transferase (GST) activity (EC.2.5.1.18):

Glutathione-S-transferase activity was determined spectrophotometrically by forming glutathione nitrobenzyl from p-nitrobenzyl chloride using glutathione as substrate. At 310 nm, the absorbance was measured against a blank. Bone GST activity was quantified as U/mg protein [49].

Bone superoxide dismutase (SOD) activity (EC.1.15.1.1):

The activity of SOD was determined by pyrogallol’s self-oxidation, which yields O_2_ at an alkaline pH (pH 8.2). The absorbance was measured at 420 nm after 30 and 90 s, and the difference between the two readings was used to calculate SOD activity, which was expressed in terms of U/mg protein [50].

Bone glutathione peroxidase (GPx) activity (EC.1.11.1.9):

Glutathione peroxidase activity is determined by subtracting the extra GSH from the total GSH in the absence of the enzyme. The assay depends on the oxidation of GSH in the presence of cumene H_2_O_2_, followed by the reaction of GSH with Ellman’s reagent, forming a yellow chromophore. The difference between the absorbance of the test and control samples was calculated at 412 nm. The bone GPx activity was expressed in the U/mg protein [51,52].

Bone reduced glutathione (GSH) level:

Reduced glutathione content is an intracellular reductant that protects the cellular components from free radicals. GSH reacts with Ellman’s reagent, creating a yellow-colored chromophore. Absorbance was measured at 412 nm with a UV spectrophotometer. The bone GSH level was assessed as mM/mg protein [53].

The total bone protein content was utilized for all assays to measure the antioxidant activity and oxidative stress biomarkers. It was measured using the Lowry method, where bovine serum albumin at 1 mg/mL concentration was used as a standard. The total bone protein content was expressed as g/dL [54].

#### 2.5.6. Molecular Analysis

a. Isolation of total RNA and quantitative real-time PCR analysis (qRT-PCR)

An easy redTM total RNA extraction kit was used to extract the femur total RNA (Enzynomics, Korea). A NanoDrop 2000 spectrophotometer (Thermo Fisher Scientific, USA) was used to quantify absorbance at 260 and 280 nm and detect the concentration and purity of RNA. Samples with a (A260/280) ratio of 1.8/2 were used. Maxime RT PreMix kit (Enzynomics, Daejeon, Republic of Korea) was used to reverse transcribe one µg of extracted RNA following the manufacturer’s protocol. cDNA was used as a template for amplification, and glyceraldehyde-3-phosphate dehydrogenase (GAPDH) as a housekeeping gene. The PCR reaction was set up by adding the following ingredients in 0.2 mL PCR tubes: 10 µL of SYBR green with low rox; 2 µL of forward and reverse primers; and 1 µL of cDNA template. The PCR tubes were then filled to the final volume of 20 µL with sterile water. Thermal cycling conditions were followed to run PCR utilizing a CFX96TM Real-Time System (BIO-RAD, USA). Initial denaturation (95 °C, 12 min), denaturation (95 °C, 45 cycles, 10 s), and annealing temperatures were variable for each primer and extension (72 °C, 20 s), as shown in Table 2. Using the 2^−∆∆Ct^ method, the target gene’s critical threshold (Ct) was standardized with the Ct of GAPDH to estimate the fold change in the target gene.

b. Western Blot Analysis

Radioimmunoprecipitation assay (RIPA) buffer was used for homogenization of femoral tissue. Phenyl methyl sulfonyl florid (PMSF), sodium florid, and EDTA were added as protease and phosphatase inhibitors. Centrifugation (13,000 rpm, 20 min, 4 °C) was utilized to separate the supernatant, and spectrophotometric analysis was used to determine the protein concentration. Equal protein quantities (100 µg) were loaded into a 10% SDS-PAGE and then blotted into the nitrocellulose membranes (Thermo Fisher Scientific, Waltham, MA, USA). The membrane was blocked with 5% BSA for an hour at room temperature, then incubated with the primary antibodies specific to HO-1 rabbit mAb (#82206), Nrf2 rabbit mAb (#12721), the phosphorylated inhibitor of nuclear factor-κB (IκB) kinase (IKK), phospho-IKKα/β (Ser176/180) rabbit mAb (#2697), NFATc1 rabbit mAb (#5861), and β-actin rabbit mAb (#4970) overnight at 4 °C. The membrane was rinsed six times for 10 min each in 1x TBST before being incubated for 2-3 hrs at room temperature with a secondary antibody anti-rabbit IgG, alkaline phosphatase-linked antibody (#7054). Nitro Blue Tetrazolium/5-Bromo4-Chloro-3-Indolylphosphate solution (Thermo Fisher Scientific, Waltham, MA, USA) was used to visualize the protein bands. Bands were quantified using GEL DOC XR+ Gel Documentation System (Bio-Rad, California, USA) and calibrated to the β-actin, a housekeeping protein, to determine the relative expression of each expressed protein.

### 2.6. Combination Index Analysis

According to Zhou, Li [55] method, the impact of the ACE and EPE mixture was described. In comparison to the single extract, the mixture of extracts may considerably produce a better (synergistic), worse (antagonistic), or no different (additive) result. This impact was calculated using the combination index (CI), which shows the synergism, antagonism, or additive [56]. The predictive value for in vitro parameters was determined using the following formula:

$$\mathrm{The}\;\mathrm{Predicted}\;\mathrm{value}\;=\left(\frac{IC50\;ACE}{2}+\frac{\mathrm{IC}50\;\mathrm{EPE}}{2}\right)$$

On the other hand, the following formula is utilized to calculate in vivo parameters:

$$The\;Predicted\;valu=\left(\frac{observed\;value\;of\;ACE}{control\;value}+\frac{\mathrm{observed}\;\mathrm{value}\;\mathrm{of}\;\mathrm{EPE}}{control\;value}\right)*control\;value$$

The formula used to determine the CI was as follows:

$$CI=\frac{Observed\;value}{Predicted\;value\;of\;combination}$$

where antagonistic, additive, or synergistic effects are indicated by a CI greater than, equal to, or less than one, respectively [55].

### 2.7. Histopathological Study 

In all studied groups, femur bone was removed from rats by disarticulating the head from the hip joint and lower condyles from the knee joint. The whole bone was decalcified in formic acid for four days and then processed into paraffin blocks. Five-micron sections were then cut using a manual rotatory microtome and stained by hematoxylin and eosin stain. The metaphysis was identified as the area right below the epiphyseal cartilage. It was assessed for pathologic changes under light microscopy; then, multiple images were taken using a microscope-adopted digital camera. At x100 power, the bone marrow was removed using paint software (Microsoft Corporation, Microsoft Windows: version 21H1(OS Build 19043.1415). The edited photos were used to calculate the surface area of bone trabeculae using Image J software (Leica application suite software, version 4.12.0) as a percentage out of the whole examined area (Bone volume/total volume; BV/TV %) [57]. Then, trabeculae bone thickness was also measured in microns (at least 20 trabeculae were assessed in each photo) [58]. Images of cortical bone at the diaphysis of the mid femur were taken at x400 power to measure the cortical bone thickness [58]. At least three fields were assessed for each parameter; then, the mean was calculated.

### 2.8. Statistical Analysis 

The values were shown as the mean ± SD. One-way ANOVA with the LSD test was used to statistically analyze differences between groups, with a *p*-value less than 0.05 considered statistically significant. This research used SPSS 16.0 (Chicago, IL, USA).

## 3. Results

### 3.1. Characterization Analysis of ACE and EPE

The research findings demonstrated that ACE and EPE include a variety of phytochemical components (phenolics, flavonoids, and alkaloids) and minerals. Compared to ACE, EPE offers a higher yield of phenolics, flavonoids, and alkaloids, but it does not contain inulin, as shown in Table 3. Generally, the elemental analysis illustrated that EPE had a greater concentration of the elements than ACE (Table 3). Na, K, Ca, P, Mg, Fe, and Al were found in tremendous amounts, but Zn, Mn, Cu, Se, Pb, Ni, Cr, and Co were found in trace amounts. 

**Figure 2:**
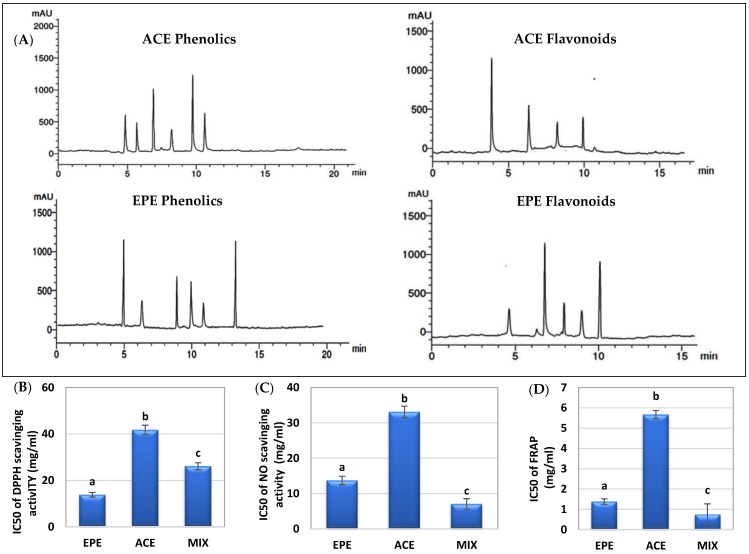
HPLC chromatograms of phenolic and flavonoid compounds in ACE and EPE (**A**). In vitro antioxidant parameters of ACE, EPE, and their MIX. (**B**) 2,2-Diphenyl-1-picrylhydrazyl (DPPH) scavenging activity. (**C**) Nitric oxide (NO) scavenging activity. (**D**) Ferric reducing antioxidant power (FRAP) scavenging activity. The results are displayed as mean ± SD. Distinct letters for the same parameter are statistically different at *p* < 0.05.

HPLC analysis in Table 4 and Figure 2A represents different levels of phenolic and flavonoid compounds in both extracts (ACE and EPE). ACE contains higher concentrations of gallic acid, cinnamic acid, naringin, and myricetin, while EPE contains syringic acid, ellagic acid, quercetin, and apigenin.

### 3.2. In Vitro Antioxidant Properties of ACE, EPE, and Their Mixture

The radical scavenging ability of EPE and ACE to DPPH, NO, and FRAP is illustrated in Figure 2B–D. EPE has a significant (*p* < 0.05) scavenging ability (lower IC50 value) higher than ACE. ACE/EPE mix showed a synergistic (CI < 1) scavenging impact on NO and FRAP, as shown in Table 5. 

### 3.3. Bone Mineral Density (BMD), Bone Mineral Content (BMC), and Bone Index

**Figure 3:**
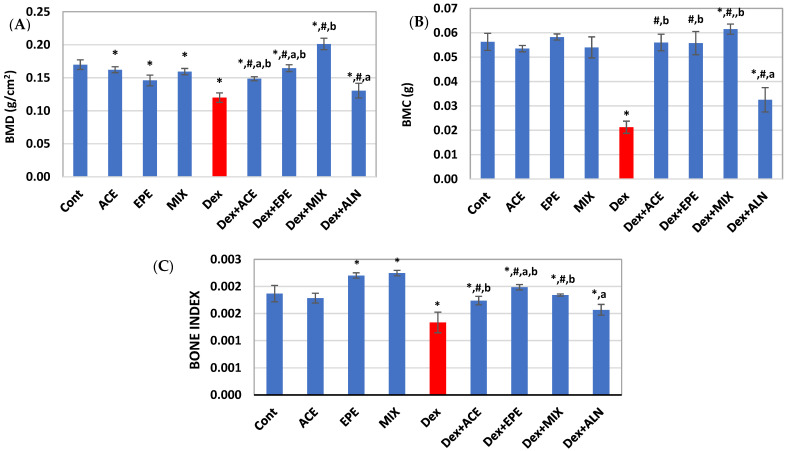
Bone quality parameters. (**A**) Bone mineral density (BMD). (**B**) Bone mineral content (BMC). (**C**) Bone index. Data are expressed as means of 6 rats for the control groups and 8 rats for the induced and the treated groups ± SD. One-way analysis of variance (ANOVA) and post hoc test (LSD) were utilized. * *p* < 0.05 against control group, ^#^ *p* < 0.05 for treated groups against Dex group, ^a^ *p* < 0.05 for treated groups against Dex + MIX group, and ^b^ *p* < 0.05 for treated groups against Dex + ALN group.

A significant decrease in BMD, BMC, and bone index was observed in the untreated Dex group compared to the control group (*p* < 0.05) (Figure 3). On the other hand, all groups that received mono or combined treatments showed a significant improvement in their bone density, content, and index. ACE/EPE MIX group showed a synergistic effect (CI < 1), as shown in Table 5. According to these findings, the combined treatment offered better validity for the treatment.

### 3.4. The Effect of Different Treatments on Serum Ionized Ca and PTH Levels

**Figure 4:**
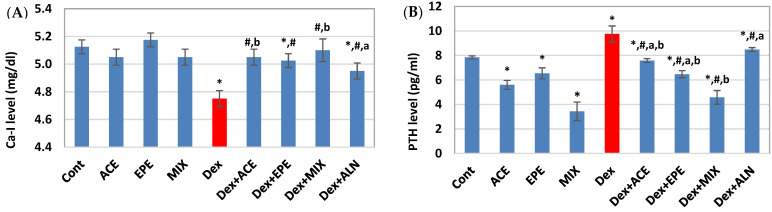
Bone biochemical analysis. (**A**) Ionized calcium (Ca-I) level. (**B**) Parathyroid hormone (PTH) level. Data are expressed as means of 6 rats for the control groups and 8 rats for the induced and the treated groups ± SD. One-way analysis of variance (ANOVA) and post hoc test (LSD) were utilized. * *p* < 0.05 against control group, ^#^ *p* < 0.05 for treated groups against Dex group, ^a^ *p* < 0.05 for treated groups against Dex + MIX group, and ^b^ *p* < 0.05 for treated groups against Dex + ALN group.

A marked elevation in serum PTH level and a significant decrease in serum ionized Ca level was noticed in the untreated Dex group compared to the control group (*p* < 0.05) (Figure 4). On the other hand, all groups who received ACE, EPE, or ACE/EPE MIX showed a significant improvement in PTH and ionized Ca-I levels compared to the Dex-injected group. ACE/EPE MIX group revealed a synergistic effect (CI < 1) in these parameters, Table 5.

### 3.5. The Effect of Different Treatments on Bone Growth and Bone Turnover Parameters

**Figure 5:**
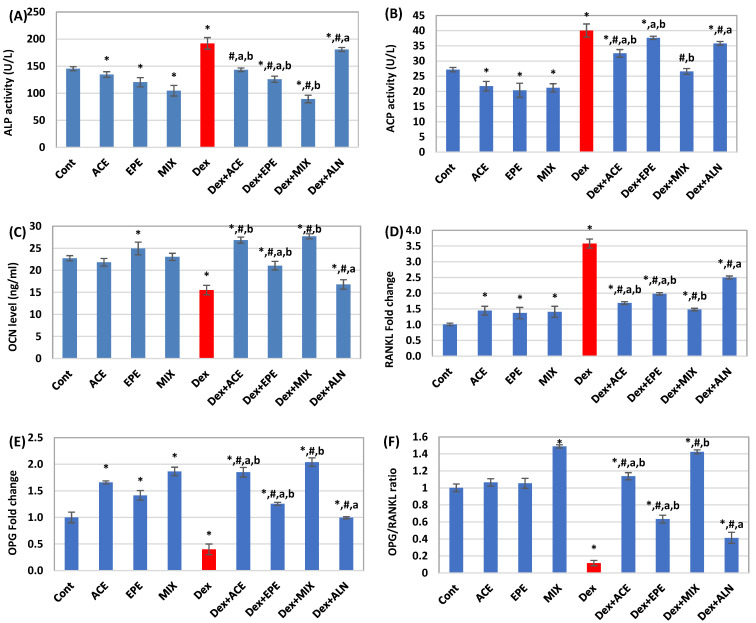

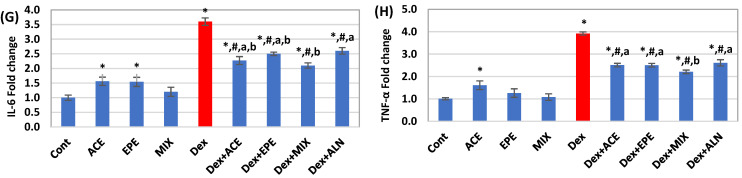
Bone formation and bone turnover parameters. (**A**) Alkaline phosphatase (ALP) activity. (**B**) acid phosphatase (ACP) activity. (**C**) Osteocalcin (OCN) level. (**D**) Receptor activator of nuclear factor kappa β ligand (RANKL) level. (**E**) Osteoprotegrin (OPG) level. (**F**) OPG/RANKL ratio. (**G**) Interleukin- 6 (IL-6) level. (**H**) Tumor necrosis factor- alpha (TNF-α) level. Data are expressed as means of 6 rats for the control groups and 8 rats for the induced and the treated groups ± SD. One-way analysis of variance (ANOVA) and post hoc test (LSD) were utilized. * *p* < 0.05 against control group, ^#^ *p* < 0.05 for treated groups against Dex group, ^a^ *p* < 0.05 for treated groups against Dex + MIX group, and ^b^ *p* < 0.05 for treated groups against Dex + ALN group.

A marked elevation in serum ALP and ACP activities associated with a significant decline in serum OCN level was noticed in the untreated Dex group compared to the control group (*p* < 0.05) (Figure 5A–C). Moreover, Dex injections resulted in a significant (*p* < 0.05) increase in the expression levels of bone RAKNL, TNF-α, and IL-6 accompanied by a significant (*p* < 0.05) decrease in OPG fold change, and the calculated OPG/RANKL ratio compared to the control group (Figure 5D–H). Administration of ACE and EPE to Dex group showed a highly significant (*p* < 0.05) decrease in ALP, ACP, IL-6, TNF-α, and RAKNL levels associated with a marked significant (*p* < 0.05) increase in OPG and OCN levels along with OPG/RANKL ratio compared to the untreated Dex group. In contrast, the group receiving Dex + ALN showed lower significant changes in these parameters than the mono- or combined-treated groups. The ACE/EPE MIX revealed a synergistic effect (CI < 1), as shown in Table 5.

### 3.6. The Effect of Different Treatments on Bone Oxidative Stress Markers

**Figure 6:**
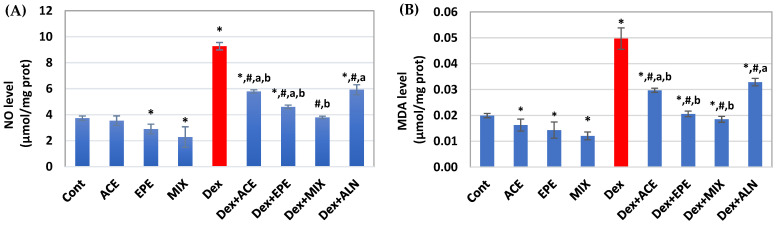

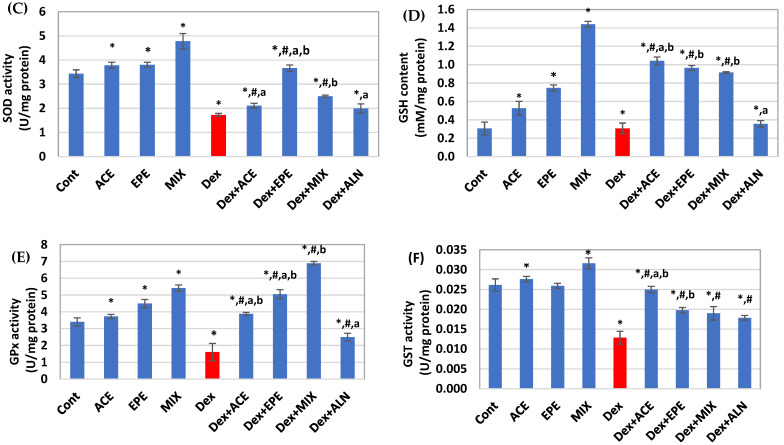
Bone oxidative stress parameters. (**A**) Nitric oxide (NO) level. (**B**) Malondialdehyde (MDA) level. (**C**) Superoxide dismutase (SOD) activity. (**D**) Glutathione (GSH) content. (**E**) Glutathione peroxidase (GPx) activity. (**F**) Glutathione S-transferase (GST) activity. Data are expressed as means of 6 rats for the control groups and 8 rats for the induced and the treated groups ± SD. One-way analysis of variance (ANOVA) and post hoc test (LSD) were utilized. * *p* < 0.05 against control group, ^#^ *p* < 0.05 for treated groups against Dex group, ^a^ *p* < 0.05 for treated groups against Dex + MIX group, and ^b^ *p* < 0.05 for treated groups against Dex + ALN group.

Figure 6 illustrated that repeated Dex injections induced oxidative stress, as illustrated by a significant elevation in bone NO and MDA levels associated with a significant (*p* < 0.05) decrease in GSH content and the activities of SOD, GPx, and GST compared to the control group. In contrast, all groups that received Dex + ACE, Dex + EPE, and MIX showed a significant (*p* < 0.05) improvement in bone NO and MDA levels, GSH content, and SOD, GPx, and GST activities compared with the untreated Dex group. ALN treated group improved these pro- and antioxidant parameters, except that SOD activity and GSH level showed a non-significant change compared with untreated Dex group. Interestingly, a synergistic (CI < 1) antioxidant effect was revealed in ACE/EPE MIX (Table 5).

### 3.7. The Effect of Different Treatments on the Protein Expression Levels of HO-1, Nrf2, NFATc1, and p-IKK

**Figure 7:**
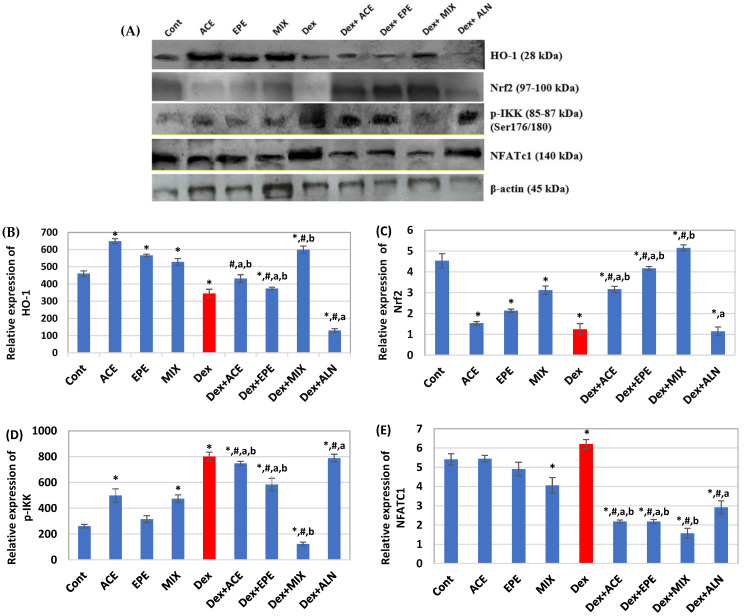
Immunoblots and relative expression levels of proteins. (**A**) Immunoblots for protein expression of Heme oxygenase-1 (HO-1), nuclear factor erythroid 2–related factor 2 (Nrf2), a phosphorylated inhibitor of nuclear factor-κB kinase p-IKKα/β (Ser176/180), nuclear factor of activated T cells 1 (NFATc1), and β-actin. (**B**) HO-1. (**C**) Nrf2. (**D**) p-IKK (Ser176/180). (**E**) NFATc1 relative expression levels. Data are expressed as mean ± SD of 3 samples/ group. One-way analysis of variance (ANOVA) and post hoc test (LSD) were utilized. * *p* < 0.05 against control group, ^#^ *p* < 0.05 for treated groups against Dex group, ^a^ *p* < 0.05 for treated groups against Dex + MIX group, and ^b^ *p* < 0.05 for treated groups against Dex + ALN group.

A significant downregulation in HO-1 and Nrf2 protein expression levels, in addition to a significant upregulation in p-IKK (Ser176/180) and NFATc1 protein levels, were observed in the Dex-untreated group compared to the control group (Figure 7). Comparing these alterations in the untreated Dex group with all treated groups, the ACE/EPE MIX treated group showed a highly significant (*p* < 0.05) increase in HO-1 and Nrf2 protein levels and a marked significance decrease in p-IKK and NFATc1 protein levels compared to ACE and EPE treated groups. On the other hand, the ALN-treated group gives lower significant (*p* < 0.05) improvement in the regulation of HO-1, Nrf2, p-IKK, and NFATc1 protein levels than the ACE and EPE groups. Interestingly, combination treatment (ACE/EPE MIX) revealed a highly synergistic effect (CI < 1), Table 5.

### 3.8. Histopathologic Outcomes and Morphometric Analysis of Experimental Groups’ Bone Tissues

**Figure 8:**
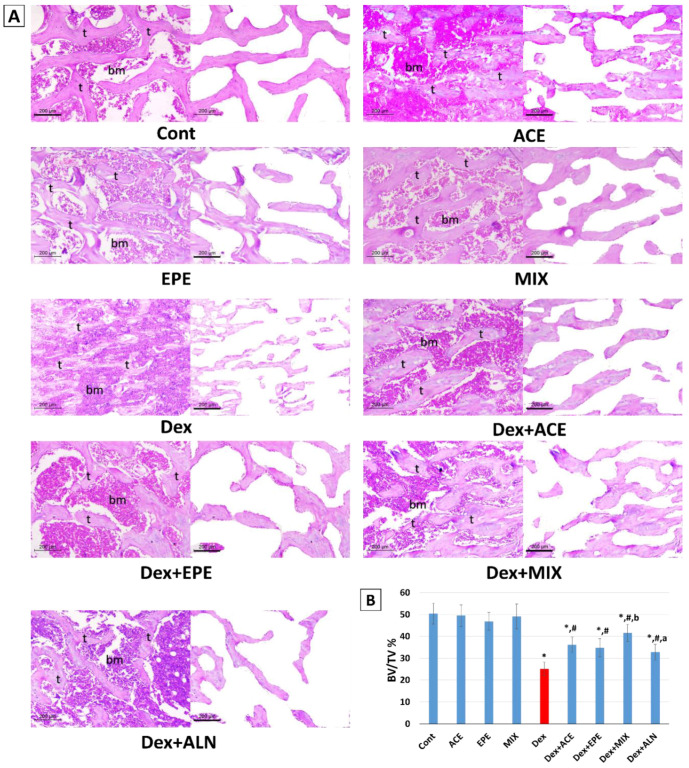
(**A**) Histopathology of the metaphysis of the upper-end femur in the studied group (x100, scale bar = 200 microns. The first photo represents the H&E-stained section to show bone trabeculae (t) and bone marrow space (bm), and the second photo reveals an edited image after the removal of bone marrow space to highlight the percentage of the trabecular bone area of total volume (BV/TV%). The Control, ACE, EPE, and MIX groups reveal a network of thick anastomosing bone trabeculae with narrow intertrabecular areas. The Dex group shows marked thinning of bone trabeculae. The BV/TV% was increased in different treated groups, with the maximum effect seen in the Dex + MIX group. (**B**) Chart of BV/TV % of different studied groups. Data are expressed as mean ± SD. One-way analysis of variance (ANOVA) and post hoc test (LSD) were utilized. * *p* < 0.05 against control group, ^#^ *p* < 0.05 against Dex group, ^a^ *p* < 0.05 against Dex + MIX group, and ^b^ *p* < 0.05 against Dex + ALN group.

**Figure 9:**
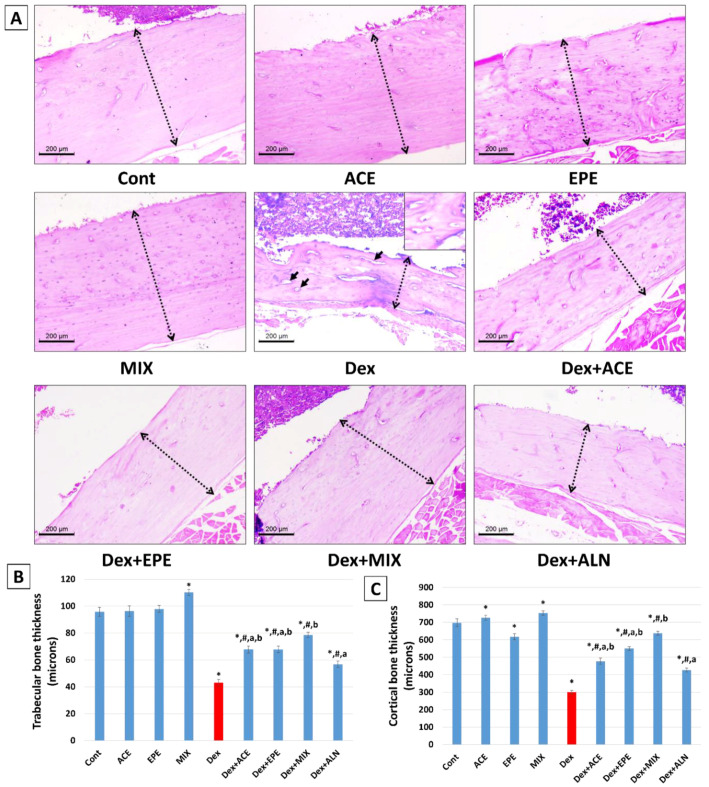
(**A**) Histopathology of cortical bone at the diaphysis of the mid-femur in studied groups (x100, scale bar = 200 microns). Dashed double-head arrows represent cortical bone thickness. The Control, ACE, EPE, and MIX groups show thick cortical bone with thin osteoblastic lining. The bone shows multiple viable osteocytes in its lacunae. The Dex group shows a markedly thinned-out cortex with an irregular surface. The bone shows multiple bone cysts (arrows) and degenerated osteocytes (inset). Those changes were reversed in different treated groups. Increased thickness is noted with the maximum effect seen in the Dex + MIX group. (**B**) Bar chart of trabecular bone thickness in different studied groups. (**C**) Bar chart of cortical bone thickness in different studied groups. Data are expressed as mean ± SD. One-way analysis of variance (ANOVA) and post hoc test (LSD) were utilized. * *p* < 0.05 against control group, ^#^ *p* < 0.05 against Dex group, ^a^ *p* < 0.05 against Dex + MIX group, and ^b^ *p* < 0.05 against Dex + ALN group.

The metaphysis of the upper-end femur in the control group shows a network of thick, irregular bone trabeculae arranged as anastomosing cords. The mean trabecular thickness was (95.7 microns). A single layer of flat osteoblasts lined all trabeculae. The bone trabeculae constituted at least 50.4% of the examined areas (BV/TV%) (Figure 8). The bone cortex in the mid-femur was thick (697.6 microns) (Figure 9). Osteoblasts covered it and contained viable osteocytes within their lacunae. The EPE, ACE, or the combined regimen of both products showed the same histology as the control group. No pathologic changes were noticed in any of them (Figure 8 and Figure 9).

The Dex group showed thinned-out, discontinuous, widely separated trabeculae (43.0 microns). They were lined with scanty osteoblasts. Wide intertrabecular areas are seen, with bone trabeculae constituting less than 25.1% of examined fields. The cortical bone was markedly thinned out (301.2 microns) with an irregularly eroded surface showing multiple resorption bits. Osteocytes showed degenerative changes in the form of wide lacunae and dark-stained nuclei (Figure 8 and Figure 9). 

Treatment with purslane and chicory in Dex + ACE and Dex + EPE groups significantly improved osteoporosis’ three assessed histomorphologic parameters. The trabecular, cortical bone thickness and BV/TV% increased to about (67.8 and 67.7 microns), (477.5 and 549.9 microns), and (36.2 and 34.8), respectively (Figure 8 and Figure 9). The combined treatment of both products in the Dex + MIX group showed maximum effect, increasing the BV/TV% to 41.6%. The bone trabeculae regained their thickness (78.5 microns). The cortical bone was thick, with increased osteoblast on the surface (637.9 microns). This was better than the Bps group, which showed only moderate improvement in osteoporosis. The BV/TV%, trabecular bone, and cortical bone thickness were 32.8%, 56.7 and 427.2 microns, respectively (Figure 8 and Figure 9).

## 4. Discussion

Glucocorticoids are widely used in various inflammatory conditions, but long-time or high doses of GCs pertain to the disturbance in bone remodeling, leading to GIO [59,60,61]. GIO is marked by rapid bone resorption followed by suppression of bone growth [6]. Therefore, patients undergoing GC therapy are advised to concurrently take anti-osteoporosis medications to prevent the onset and progression of GIO [7]. The current study investigated the preventative and synergistic effect of purslane and chicory extracts, their combination on GIO compared with bisphosphonate treatment (Alendronate), and their possible mechanism in preventing pathological bone destruction.

Calcium and phosphate are essential components of the inorganic matrix of bone and are vital for maintaining bone health. Their deficiency can lead to bone pathology and clinical illness [62]. In addition, BMD, BMC, and bone index are significant parameters commonly used to assess bone quality and strongly correlate with osteoporosis severity [8]. Dex injections significantly decreased these bone parameters by decreasing calcium content. This decline was reported in several studies on GC administration [12,63]. GCs can indirectly influence bone and promote osteoporosis by reducing intestinal absorption of calcium and increasing renal loss. Alterations in calcium homeostasis result in hyperparathyroidism, increasing osteoclast activity [7]. The elevated serum PTH and the declined serum calcium levels in untreated Dex rats are compatible with the findings of Canalis and Giustina [61], Wang, Yang [8], and Abdelfattah, Mohamed [7]. 

Serum total ALP and OCN are strong predictors of bone loss [62]. One of the most common isoforms of ALP is bone-specific ALP (BALP), which is physiologically attached to the osteoblastic cell membrane with only a limited amount leaked into the serum. Mukaiyama, Kamimura [64] reported a strong correlation between total ALP and BALP. OCN is a non-collagenous protein that binds to calcium and, consequently, hydroxyapatite, working to regenerate bone tissue. Generally, serum ALP activity reflects bone turnover and fracture risk, and OCN level reflects the process of bone growth [65,66]. Repeated Dex injections resulted in a significant elevation in ALP activity and a significant decline in OCN level, which agrees with the findings of earlier studies, indicating decreased osteogenesis and eventual osteonecrosis [10,44,67,68]. During the GC treatment, osteocyte apoptosis starts, and bone loss occurs. GCs reduce the osteoblasts by preventing their replication and differentiation and enhancing the apoptosis of mature osteoblasts [69]. Further, oxidative stress is a well-known risk factor that promotes apoptosis of osteoblasts and osteocytes, releasing its contents in the surroundings. It also elucidates the elevated ALP activity in the untreated Dex group [70].

Osteoblasts release RANKL and OPG, which regulate its differentiation. RANKL is a cytokine essential for osteoclast differentiation, and its expression is induced by bone-resorbing factors such as PTH, IL-6, and 1α,25-dihydroxy vitamin D3 [13]. The binding of RANKL to RANK sends signals to cells through tumor necrosis factor receptor-associated factors (TRAFs). RANKL/RANK/TRAF6 signaling activates the IKK/ NF-κB pathway [9], which promotes the expression level of osteoclastogenic factors, such as NFATc1, a master transcription factor of osteoclast differentiation. Subsequently, it activates osteoclast-related genes such as ACP, a mediator that contributes to the differentiation of osteoclast progenitor cells into osteoclasts [12,13]. ACP is usually overexpressed in osteoclasts, disrupting bone tissue and developing osteoporosis [71]. On the contrary, OPG acts as a soluble decoy receptor for RANKL, counteracting its effects on bone by inhibiting its interaction with RANK [13,72]. Consistent with these findings, our results revealed that Dex injections promote osteoclastogenesis via decreasing OPG levels and subsequently enhanced cellular induction of RANKL, NFATc1, and ACP levels along with diminished OPG/RANKL ratio. This provides insight into the mechanism contributing to the early onset of bone resorption in GIO. These findings agree with the studies of Tobeiha, Moghadasian [14], Kondo, Kitazawa [15]. Furthermore, RANKL/RANK interaction, which can promote NF-κB activation, configures interesting molecular links between bone remodeling and inflammation.

Studies indicate that active and systemic bone inflammation increases osteoporosis and osteoporotic fracture risk [73]. Apoptotic osteocytes stimulate the discharge of inflammatory cytokines such as IL-6 and TNF-α that initiate osteoclastogenesis [8]. The transcription factor NF-κB intensifies the first inflammatory reaction that induces the release of proinflammatory cytokines. NF-κB stays as an inactive complex with its inhibitory IκB proteins in the cytoplasm. NF-κB activation results from the phosphorylation of IκB by specific IκB kinases (IKKs) on two conserved serine residues near its N-terminus [74]. The IKK-dependent phosphorylation of IκB marks it for ubiquitination and proteasomal degradation, liberating NF-κB. NF-κB translocated to the nucleus, which binds DNA and promotes the transcription of target proinflammatory molecules, such as TNF-α and IL-6. In turn, TNF-α stimulates a cascade of intracellular reactions that leads to the activation of IKK, resulting in the phosphorylation of IκB and the subsequent release and translocation of NF-κB to the nucleus [74]. In addition, TNF-α was reported to affect bone formation by inhibiting osteoblast differentiation, reducing collagen synthesis by osteoblasts, and promoting osteoblast apoptosis. Furthermore, IL-6 and TNF-α induce RANKL, which drives osteoclastogenesis [75]. Consistent with these studies, our data suggest that Dex induced NF-κB activation through increasing phosphorylation of IκB kinases (IKKα) and subsequently enhanced the expression levels of the proinflammatory mediators, TNF-α and IL-6, which are critical for osteoclast differentiation. These findings illustrated the indirect proinflammatory effect of Dex through RANKL/RANK interaction. These findings are compatible with Han, Zhang [73]. Therefore, IKK inhibitors can be a promising therapy to improve fracture healing in osteoporosis patients.

**Figure 10:**
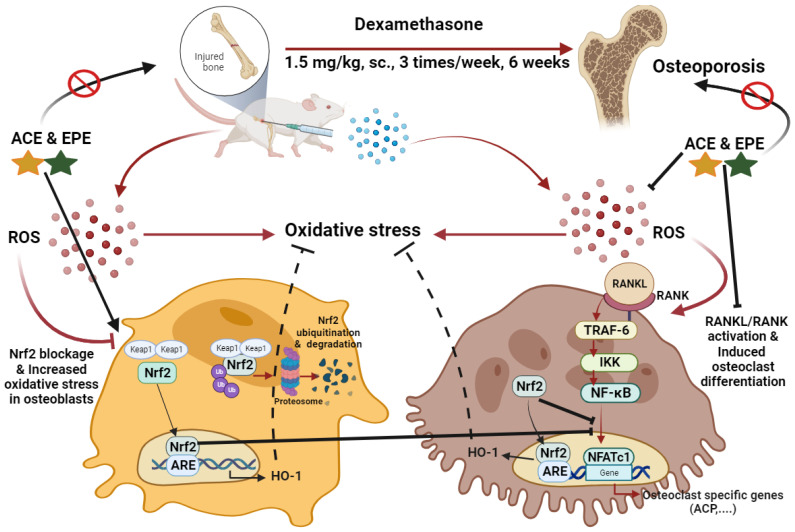
Mechanisms of glucocorticoid-induced osteoporosis and possible treatment of ACE and EPE. Dex initiates oxidative stress through the accumulation of ROS. ROS activates RANKL/RANK/TRAF6 signaling that potentiates the IKK/NF-κB pathway, NFATc1 expression, and osteoclast-specific genes, including ACP activity. At the same time, Dex blocked Nrf2/HO-1 activation. Keap1 sequesters Nrf2 and facilitates its ubiquitination and proteasomal degradation in the cytoplasm. However, the cytoprotective potential under oxidative stress starts from Keap1 detachment from Nrf2, facilitates Nrf2 nuclear translocation, modulates ARE-dependent gene expression, and induces HO-1 expression responsible for ROS and oxidative stress scavenging. Activated RANKL/RANK signaling and suppressed Nrf2/HO-1 signal activated osteoclast differentiation and induced bone resorption, a leading cause of the development and initiation of osteoporosis. ACE and EPE represent antioxidant and anti-inflammatory actions that suppress bone resorption and stimulate bone formation through the activation of Nrf2/HO-1 signal and the blockage of RANKL/RANK activation. Red arrows represent Dex action, while black ones illustrate the possible protective effect of ACE and EPE.

Glucocorticoids-induced osteoporosis is closely related to oxidative stress [8]. Glucocorticoids have been shown to induce oxidative stress indirectly by inhibiting antioxidant enzymes or depleting antioxidant molecules. Studies showed that Dex significantly diminished the antioxidant defense systems via depleting the Nrf2 signaling pathway and its downstream agents, including the HO-1 [24]. The Nrf2/HO-1 pathway is crucial in oxidative stress-fighting in bone cells. Under normal or non-stress conditions, Nrf2 is lowly expressed and localized in the cytoplasm bound to its sequestering protein, Kelch-like ECH-associated protein 1 (Keap1). The interaction between Keap1 and Nrf2 prevents the nucleus translocation of Nrf2 and facilitates its ubiquitination and proteasomal degradation. However, under oxidative stress, the cysteine residues that maintain the Keap1 association with Nrf2 are oxidized, modifying their shape and releasing Nrf2. Unbound Nrf2 translocated to the nucleus, modulating ARE-dependent gene expression and initiating the cytoprotective signaling cascade [19,20] (Figure 10). 

HO-1 possesses anti-inflammatory, antioxidative stress, and anti-apoptosis properties [76]. Moreover, HO-1 is essential to sustain bone homeostasis, as it plays a considerable role in promoting osteoblast differentiation and suppressing osteoclast differentiation, making it a potential target for osteoporosis treatment [77]. A significant decline in Nrf2 and HO-1 was noticed in untreated Dex rats, which is in agreement with Xu, Guan [16], Sun, Xu [22], who deduced that Dex induces oxidative stress by inhibiting the activation of antioxidant systems and Nrf2 deficiency, promotes osteoclast proliferation and bone resorption. Moreover, Dex elevated MDA and NO levels, diminished bone GSH content, and reduced GST, GPx, and SOD activities. These results indicate the imbalance between the Dex group’s prooxidants and antioxidative defense system, which is compatible with the results of previous studies [44,78,79]. Further, oxidative stress enhances the apoptosis of osteoblasts [80]. Moreover, oxidative stress stimulated the RANKL/RANK interaction while inhibiting the RANKL/OPG axis, which promotes osteoclast differentiation. 

The assessed histological parameters align with observed biochemical and molecular findings. Trabecular and cortical bone thicknesses and BV/TV% showed thinned, discontinuous, widely separated trabeculae lined with scanty osteoblasts, thinned out irregular eroded cortical surface with degenerative osteocytes along with low bone trabecular area in the Dex group compared to the control group. These histological changes are compatible with the findings of [10,69] that showed a higher degree of microarchitectural deterioration, bone loss, and decreased bone formation in rats upon Dex injection. From these findings, we can conclude that repeated Dex injections induced an osteoporotic model.

Traditionally, many medications have been used to prevent GIO. Bisphosphonates (Bps) are anti-resorptive agents and are currently the recommended therapy and a crucial strategy for lowering GCs-induced bone fragility and osteoporosis [14]. Alendronate (ALN) is a member of the Bps pharmacological class [81]. ALN binds hydroxyapatite crystals, suppresses osteoclast-mediated bone resorption, and reduces the breakdown of bone matrix. ALN regulates mineral reabsorption and decreases bone turnover rate [82]. However, long-term ALN therapy is associated with severe side effects, resulting in reduced or absent osteoblastic surface, delayed healing, and increased risks of atypical fractures, although BMD is not significantly decreased [83]. In the present study, ALN ameliorated the abovementioned changes in the Dex-induced model with variable degrees, with a lower impact than ACE, EPE, and ACE/EPE MIX. ALN improves bone quality by increasing BMD and BMC, enhancing serum Ca levels, and reducing PTH levels.

Moreover, the present data also suggest that ALN affected bone turnover and growth, as evidenced by decreased ALP activity and increased OCN levels. Further, the data suggest that ALN regulates antioxidant and oxidative stress parameters while limiting the inflammatory response. This is supported by its partial inhibition of IKK activation, which is pivotal for NF-κB activation, inflammation, and osteoclastogenesis [84]. Through the current results, ALN decreased MDA, NO, TNF-α, IL-6, and p-IKK levels associated with elevated GPx and GST activities. These data are suitable with the findings of Chakraborty and Dhara [85] for oxidative stress parameters and Papadaki, Tsatsanis [86] for reduced inflammatory cytokines. Incompatible with our findings, Wang, Yang [8] revealed that ALN treatment significantly boosted serum Ca and OCN levels with reduced PTH, ACP, ALP, and MDA levels and alleviated TNF-α, IL-1β, and IL-6 levels.

Additionally, ALN inhibited osteoclast formation and its resorption function by regulating the RANKL/OPG axis [87,88]. Wei, PENG [89] and Sheng, Lao [90] also illustrated that ALN strongly suppressed bone resorption directly by inhibiting RANKL and NFATc1 expression. In the current study, ALN markedly decreased RANKL and NFATc1 expression while increasing OPG expression and elevating OPG/RANKL ratio. In addition, Chakraborty and Dhara [85] reported that ALN attenuated GIO by moderately increasing cortical and trabecular bone thickness and eliminating osteoporotic cavities. These detailed histopathological examinations are compatible with our findings. However, Bps were described as relatively weak modulators that do not completely rescue GIO and do not appear effective in preventing fragility fractures [75]. This opens the door for novel strategies that optimize bone strength in GCs-treated patients while minimizing the side effects. Recently, natural products have been relied upon instead of traditional treatment due to their safety and effectiveness. 

The current study evaluated the antiosteoporotic effect of two Egyptian edible plants (purslane and chicory) against GIO. The osteoprotective effect of chicory and purslane extracts and their combination is still not fully explored. Chicory and purslane extracts are a source of natural antioxidants and valuable bioactive compounds [28,31,91]. The in vitro results assessed that ACE and EPE are rich in phytochemicals, essential minerals, and potent antioxidants. Phenolics possess anti-inflammatory, antioxidant, and antiapoptotic potential [92]. Flavonoids are a group of naturally occurring chemicals that have been shown to effectively counter osteoporosis by stimulating bone formation [93]. Also, alkaloids are potent antioxidant, antiresorptive, and anti-osteoporotic agents [94]. Our recent study [28] investigated that chicory and purslane are polyphenol- and vitamin-rich foods. ACE has a large amount of gallic acid, cinnamic acid, naringin, and apigenin. At the same time, EPE contains flavonoids, such as quercetin, myricetin, naringin, and plenty of phenolic acids, including syringic acid, ellagic acid, gallic acid, and pyrogallol. Moreover, ACE and EPE contain adequate amounts of vitamin C and A, respectively. These vitamins and polyphenols are potent antioxidants. Further, DPPH, NO, and FRAP radical scavenging activity of ACE [95,96] and EPE [97,98,99] were previously reported.

Moreover, essential minerals, such as selenium, manganese, copper, zinc, and iron, are essential for a group of antioxidant enzymes (GPx, SOD, and catalase) participating in the antioxidant defense system against oxidative stress [100]. Otherwise, the bone stores about 60% of the overall amount of magnesium, and its deficiency contributes to osteoporosis directly by acting on crystal formation and indirectly by affecting PTH. Also, hypomagnesemia enhances inflammation by promoting inflammatory genes such as TNF-α and IL-6, which suppress osteoblast function and promote osteoclast function [101]. Subsequently, the cortical bone thickness was significantly reduced in the Dex group and recovered upon ACE and EPE administration. Calcium and phosphorus are essential in bone’s structural integrity and maintaining a complex bone matrix. Approximately 99% of the body’s calcium and about 85% of the body’s phosphorus are found in the bone as hydroxyapatite. The Ca/P ratio is vital for bone formation, so a low Ca/P ratio affects calcium homeostasis, consequently increasing the risk of bone shatter and osteoporosis [102]. Moreover, higher dietary potassium concentration is significantly associated with increased BMD and a reduced risk for osteoporosis [103]. 

In the current study, daily administration of ACE and EPE as mono or combined therapy before Dex injections significantly improved the serum Ca level, BMD, BMC, and bone index compared to the untreated Dex group. This protective effect is primarily due to the high mineral content in both ACE and EPE. Moreover, purslane was reported to contain high calcium content [28], which is essential for improving these bone parameters and normalizing the PTH level. Furthermore, chicory root is the most plentiful source of dietary fiber inulin, which is reported to enhance calcium absorption, improve femur and tibia mineral contents, and increase whole-body BMD in growing male rats [104]. Therefore, ACE can fight osteoporosis by increasing intestinal Ca absorption and retaining PTH to its normal level due to its high inulin content [44]. ACE/EPE MIX administration revealed a synergistic effect in these parameters. 

The protective effect of ACE and EPE on bone loss is mainly due to the suppression of osteoclast differentiation and function and enhanced osteoblastic bone formation. During osteoporosis, the activity of bone parameters is altered. ACE and EPE remarkably reversed this effect. ACE and EPE significantly decreased bone turnover and fracture risk parameters (ALP activity) and increased osteoblastogenesis biomarker (OCN level). Also, the current study showed that ACE and EPE dampened cellular induction of the proinflammatory cytokines (IL-6 and TNF-α) and IKK phosphorylation, which are critical players in osteoclast differentiation. The anti-osteoclastogenic action of ACE and EPE could be due to their potential anti-inflammatory function. Previous studies support the anti-inflammatory potential of chicory [105,106] and purslane [107,108] extracts.

Additionally, the osteoprotective and inhibitory effects of ACE and EPE on osteoclast differentiation were confirmed by inhibiting RANKL/RANK interaction through upregulation of OPG levels, OPG/RANKL ratio, and depleted RANKL level. Moreover, ACE and EPE firmly blocked the RANKL-induced activation of NFATc1, and subsequently, ACP activity was reduced. These results are consistent with Kim, Oh [109], who verified the anti-osteoclastogenic activity of EPE for the first time. EPE suppressed osteoclast differentiation and bone resorbing activity by inhibiting Akt/GSK3β-c-Fos-NFATc1 signaling cascades and preventing lipopolysaccharide-induced osteolysis and bone loss [109]. Concerning ACE-suppressed RANKL-induced NFATc1 expression and inhibited osteoclastogenesis, these findings demonstrate the first insight into the mechanism of ACE osteoprotection. 

ACE and EPE remarkably altered the level of antioxidant enzymes modulated during the Dex-induced osteoporosis. ACE and EPE restored the expression level of Nrf2 and HO-1, declined MDA and NO levels, and caused a discernible elevation of GSH content and the activities of SOD, GST, and GPx. These were compatible with the studies that confirmed that EPE [108,110] and ACE [28,105,111] are potent antioxidants that are secure and efficient in improving oxidative state. 

Consistent with the biochemical results, ACE and EPE restored the trabecular connectivity, bone strength, and bone volume by increasing the trabecular and cortical bone thicknesses and BV/TV%. Moreover, microarchitecture analysis revealed a powerful effect of the ACE/EPE combination by restoring bone constituents. ACE/EPE combined therapy effectively inhibited GIO by modulating the estimated signaling cascades. According to the combination index values, this combined therapy displayed synergistic activity (CI < 1) in most examined parameters. Mixing the ACE and EPE increases their impact and results in boosted downregulation of the RANKL/RANK/NFATc1 pathway, enhanced anti-inflammatory and antioxidant activities that inhibit the osteoclast activity, and improved osteoblastic cell function. This may be due to gathering their phytochemicals, minerals, and vitamins. Taken together, by using an empirical model of Dex-induced osteoporosis, our investigation suggested the synergistic effects of combining ACE and EPE on bone quality, Ca homeostasis system, bone formation markers, bone turnover markers, osteoclastogenesis axis, antioxidant defense system, and histological assessment. ACE and EPE are considered multitarget natural medicines that control the pharmacological response of numerous pathways and may help treat bone-related diseases and the fight against GIO.

## 5. Conclusions

Dex injection led to bone erosion due to accumulated free radicals and initiated oxidative stress, increased RANKL/RANK interaction, NFATc1 expression, and ACP activity. On the other hand, this is the first report that verifies the osteoprotective potential of chicory root extract and its combination with purslane leaf extract as two Egyptian salad plants and their possible mechanisms of action. ACE and EPE prevented Dex-induced bone loss by stimulating the Nrf2/HO-1 pathway and inhibiting RANKL-induced osteoclast differentiation (Figure 10). Therefore, it was found that ACE and EPE suppressed bone resorption and stimulated bone formation, which may be due to their phytochemical content, minerals, inulin, and potent antioxidant and anti-inflammatory activity. In contrast, ALN reduced Dex-induced bone loss merely by suppressing bone resorption. However, these findings need more and more work to confirm the exact mechanism by which ACE and EPE exert their osteoprotective potential. This may be achieved by using Nrf2 knockout animals or in vitro testing to dissect the RANKL versus antioxidant response pathways.

In conclusion, herbal medication is commonly used instead of chemical drugs because of its powerful components and minor side effects. So, from a clinical standpoint, mixing ACE and EPE with GC therapy may show improved results in preventing or treating GIO. Taken together, paying attention to the minerals and nutrients in the diet is very important.

## Figures and Tables

**Table 1:** Plant material collection and extract preparation.

Plant Name/Family	Used Part	Place and Time of Collection	Used Solvent	Weight of Dry Powder
Chicory (*Cichorium intybus* L.)/*Asteraceae*	Roots	Egyptian farmers (Kafr El Dawar, El Beheira, Egypt)/winter (2019)	Water	500 g
Purslane (*Portulaca oleracea* L.)/*Portulacaceae*	Leaves		Ethanol (70%)	300 g

**Table 2:** Primers’ sequence and qRT-PCR conditions.

Gene Name/Accession Number	Primers’ Sequence		Annealing Temp. (°C)
	Forward	Reverse	
GAPDH/NM_017008.4	TCCCTCAAGATTGTCAGCAA	AGATCCACAACGGATACATT	52
OPG/NM_012870.2	GTTCTTGCACAGCTTCACCA	AAACAGCCCAGTGACCATTC	54
RANKL/NM_057149.2	ACCAGCATCAAAATCCCAAG	GGCCGCTAATTTCCTCACCA	52
TNF-α/NM_012675.3	ACACACGAGACGCTGAAGTA	GGAACAGTCTGGGAAGCTCT	62
IL-6/NM_012589.2	GCCAGAGTCATTCAGAGCAATA	GTTGGATGGTCTTGGTCCTTAG	55

GAPDH, glyceraldehyde-3-phosphate dehydrogenase; OPG, osteoprotegerin; RANKL, receptor activator of nuclear factor kappa β ligand; TNF-α, tumor necrosis factor-alpha; IL-6, Interleukin-6.

**Table 3:** Phytochemical parameters and mineral contents of EPE and ACE.

Parameter	ACE Content	EPE Content
Yield (g%)	10.32 ± 0.41	16.61 ± 0.32
Phytochemical constituents		
Total Phenolics (mg gallic acid Eq/g extract)	21.61 ± 0.51	31.86 ± 0.61
Total flavonoids (mg quercetin Eq/g extract)	103.91 ± 1.74	111.36 ± 3.6
Total alkaloids (%)	22.68 ± 0.14	31.73 ± 1.2
Inulin content (g%)	22.75 ± 0.18	-
Minerals content (µg/mg extract)		
Sodium	147.087	1339.584
Potassium	677.287	136.755
Calcium	95.840	682.019
Phosphorus	97.842	432.704
Magnesium	21.589	601.108
Iron	21.967	74.395
Aluminum	5.155	32.612
Zinc	0.694	3.671
Manganese	0.508	1.635
Cupper	0.645	1.317
Selenium	0.015	0.030
Lead	0.077	0.040
Nickel	0.035	0.151
Chromium	0.278	0.308
Cobalt	0.01	0.029

**Table 4:** HPLC analysis of phenolic and flavonoid compounds in ACE and EPE.

Phenolic Compounds (mg/g Extract)			
RT#	Compound	ACE	EPE
5.0	Syringic acid	1.082	2.850
5.8	p-Coumaric acid	0.824	0.872
7.0	Cinnamic acid	2.444	ND
8.1	Caffeic acid	0.636	ND
9.0	Pyrogallol	ND	1.638
9.8	Gallic acid	3.330	1.490
10.7	Ferulic acid	1.228	0.620
13.0	Ellagic acid	ND	2.592
Flavonoid Compounds (mg/g extract)			
4.0	Naringin	3.078	1.300
6.2	Myricetin	1.844	ND
7.0	Quercetin	ND	2.940
8.0	Kampferol	1.038	1.022
9.1	Luteolin	ND	0.798
10.0	Apigenin	1.556	2.548

ND not detected.

**Table 5:** Combination index of the tested parameters.

Parameter	CI	Effect
DPPH scavenging (mg/mL)	0.939 ± 0.1	Additive
NO scavenging (mg/mL)	0.301 ± 0.12	Synergistic
FRAP (mg/mL)	0.215 ± 0.28	Synergistic
BMD (g/cm^2^)	0.642 ± 0.01	Synergistic
BMC (g)	0.547 ± 0.16	Synergistic
Bone index	0.500 ± 0.02	Synergistic
Ca-I (mg/dl)	0.506 ± 0.01	Synergistic
PTH (pg/mL)	0.326 ± 0.06	Synergistic
OCN (ng/mL)	0.578 ± 0.21	Synergistic
ACP activity (U/L)	0.359 ± 0.598	Synergistic
ALP activity (U/L)	0.332 ± 0.63	Synergistic
TNF- α fold change	0.306 ± 0.01	Synergistic
IL-6 fold change	0.389 ± 0.02	Synergistic
OPG fold change	0.588 ± 0.02	Synergistic
RANKL fold change	0.254 ± 0.01	Synergistic
OPG/RANKL ratio	0.859 ± 0.02	Synergistic
NO level (µmol/mg protein)	0.365 ± 0.12	Synergistic
MDA level (µmol/mg protein)	0.353 ± 0.001	Synergistic
GSH content (mM/mg protein)	0.359 ± 0.01	Synergistic
GPx activity (U/mg protein)	0.772 ± 0.05	Synergistic
SOD activity (U/mg protein)	0.434 ± 0.08	Synergistic
GST activity (U/mg protein)	0.422 ± 0.001	Synergistic
HO-1 relative expression level	0.747 ± 0.31	Synergistic
Nrf2 relative expression level	0.703 ± 0.01	Synergistic
p-IKK relative expression level	0.091 ± 0.05	Synergistic
NFATc1 relative expression level	0.388 ± 0.03	Synergistic

## Data Availability

Data is contained within the article.
